# Supplementary material for: Optimization of SNAP-25 and VAMP-2 Cleavage by Botulinum Neurotoxin Serotypes A–F Employing Taguchi Design-of-Experiments
Source: Toxins (Basel). 2019 Oct 11;11(10):588. doi: 10.3390/toxins11100588 (PMC6832249; doi:10.3390/toxins11100588)
Supplement: Supplementary file 1 [file toxins-11-00588-s001.pdf]

toxins

MDPI

Supplementary Materials: Optimization of SNAP-25 and VAMP-2 Cleavage by Botulinum Neurotoxin Serotypes A–F Employing Taguchi Design-of-Experiments

Laura von Berg, Daniel Stern, Jasmin Weisemann, Andreas Rummel, Martin Bernhard Dorner and Brigitte Gertrud Dorner

Table S1. Selected publications analyzing factors influencing BoNT substrate cleavage.

Toxin/ LC	Substrate	Substances Tested	Method	Key Findings	Reference
LC/A<br>LC/B<br>LC/E	SNAPtide (List laboratories)	BSA, TMAO, other osmolytes/crow-ding agents (proline, betain, glycerol, ficol)	FRET (multiwell fluorescence assay using SNAPtide)<br>HPLC	BSA increases LC/A activity; TMAO strongly increases LC/A, LC/B, and LC/E activity	[1]
BoNT/A<br>BoNT/E	SNAP-25 (137-206) (ELISA)<br><br>SNAP-25 (1-206) (Western blots)	DTT, NaCl, Tween20, BSA	ELISA (with neoepitope specific polyclonal antibodies), Western blots	Tween20 (<0.5%) increases BoNT/A activity;<br>BSA slightly increases BoNT/A activity;<br>2.5 mM DTT optimal for BoNT/E;<br>5 mM optimal for BoNT/A;<br>NaCl leads to reduced BoNT/E activity	[2]
LC/A	66-mer: SNAP-25 (141-206)<br><br>17-mer: SNAP-25 (186-203)<br><br>SNAP-25 (1-206)	Different substrate lengths, NaCl, DTT, BSA, $ZnCl_2$	UPLC/HPLC	Effects of different factors depend on substrate lengths;<br>NaCl (<100 mM) increase LC/A activity with full-length substrate;<br>$ZnCl_2$ (<120 µM), DTT (<4 mM) increase LC/A with full-length substrate;<br>BSA doubles LC/A activity	[3]
BoNT/E	H6-SNAP-25	Trypsin digest (nicking), reducing conditions	SDS-PAGE	Reducing conditions improve endopeptidase activity of BoNT/E;<br>nicking improves BoNT/E activity	[4]
BoNT/B<br>LC/B	h/rVAMP-2 (60-94)	Trypsin, pH, HEPES-, Pipes-, phosphate buffer, NaCl, divalent cations (Zn^{2+} etc), Asolectin	SDS-PAGE	Optimal activity of BoNT/B at pH 7.2;<br>highest activity of BoNT/B observed in HEPES buffer;<br>NaCl strongly reduced BoNT/B activity	[5]
BoNT/A (List Labs)	SNAP-25 (187-201)	BSA, other species serum albumin	HPLC	2 mg/ml BSA increased activity of BoNT/A;<br>other serum albumins with slightly stimulating effect	[6]
LC/B<br>LC/E	VAMPtide	Temperature, folding properties	FRET	LC/B and LC/E optimally active at 37 °C	[7]

Table S2. Results of the ANOVA of L9-Array Experiments 1 and 2 of BoNT/A.

Factor	DF	SQ	V	F-Value	p [%]	*p* Value
pH	2	*11*	5.4	1.23	10	0.384
$ZnCl_2$	2	*7*	3.4	0.77	6	0.520
DTT	2	69	34.3	7.86	61	**0.041**
NaCl	2	26	12.8	2.93	23	0.164
BSA	2	134	67	3	15	0.144
TMAO	2	688	344	17	76	**0.011**
Tween 20	2	*47*	23	1	5	0.405
empty (control)	2	*35*	18	1	4	0.490

DF = Degrees of freedom; SQ = Sumsquares; V = Variance. Italic numbers indicate values used for error estimation; bold numbers indicate significant factors ($p < 0.05$).

Table S3. Results of the ANOVA of L9-Array Experiments 1 and 2 of BoNT/B.

Factor	DF	SQ	V	F-Value	p [%]	*p* Value
pH	2	*4*	2	0	3	0.750
$ZnCl_2$	2	*24*	12	2	18	0.294
DTT	2	68	34	5	52	0.086
NaCl	2	35	17	2	27	0.199
BSA	2	*0*	0	0	0	0.947
TMAO	2	38	19	18	57	**0.010**
Tween 20	2	24	12	12	37	**0.021**
empty (control)	2	*4*	2	2	6	0.257

DF = Degrees of freedom; SQ = Sumsquares; V = Variance. Italic numbers indicate values used for error estimation; bold numbers indicate significant factors ($p < 0.05$).

Table S4. Results of the ANOVA of L9-Array Experiments 1 and 2 of BoNT/C.

Factor	DF	SQ	V	F-Value	p [%]	*p* Value
pH	2	34	17	2	22	0.25
$ZnCl_2$	2	86	43	5	56	0.08
DTT	2	*32*	16	2	21	0.27
NaCl	2	*2*	1	0	2	0.88
BSA	2	3	1	10	25	**0.028**
TMAO	2	8	4	27	70	**0.005**
Tween 20	2	*1*	0	2	5	0.255
empty (control)	2	*0*	0	0	0	0.962

DF = Degrees of freedom; SQ = Sumsquares; V = Variance. Italic numbers indicate values used for error estimation; bold numbers indicate significant factors ($p < 0.05$).

Table S5. Results of the ANOVA of L9-Array Experiments 1 and 2 of BoNT/D.

Factor	DF	SQ	V	F-Value	p [%]	*p* Value
pH	2	*28*	14	1	10	0.43
$ZnCl_2$	2	147	74	5	51	0.07
DTT	2	*26*	13	1	9	0.45
NaCl	2	85	42	3	30	0.15
BSA	2	*2*	1	0	1	0.673
TMAO	2	229	115	48	93	**0.002**
Tween 20	2	7	4	2	3	0.315
empty (control)	2	*7*	4	2	3	0.315

DF = Degrees of freedom; SQ = Sumsquares; V = Variance. Italic numbers indicate values used for error estimation; bold numbers indicate significant factors ($p < 0.05$).

Table S6. Results of the ANOVA of L9-Array Experiments 1 and 2 of BoNT/E.

Factor	DF	SQ	V	F-Value	p [%]	*p* Value
pH	2	*12*	6	1	7	0.40
$ZnCl_2$	2	87	43	9	52	**0.04**
DTT	2	60	30	6	36	0.06
NaCl	2	*8*	4	1	5	0.50
BSA	2	58	29	1	8	0.431
TMAO	2	557	279	10	77	**0.027**
Tween 20	2	*54*	27	1	7	0.452
empty (control)	2	*56*	28	1	8	0.437

DF = Degrees of freedom; SQ = Sumsquares; V = Variance. Italic numbers indicate values used for error estimation; bold numbers indicate significant factors ($p < 0.05$).

Table S7. Results of ANOVA of L9-Array Experiments 1 and 2 of BoNT/F.

Factor	DF	SQ	V	F-Value	p [%]	*p* Value
pH	2	*24*	12	1	14	0.35
$ZnCl_2$	2	36	18	2	21	0.24
DTT	2	99	50	6	58	0.07
NaCl	2	*11*	6	1	7	0.58
BSA	2	7	4	7	32	**0.049**
TMAO	2	14	7	13	60	**0.017**
Tween 20	2	*1*	1	1	6	0.351
empty (control)	2	*1*	0	1	3	0.581

DF = Degrees of freedom; SQ = Sumsquares; V = Variance. Italic numbers indicate values used for error estimation; bold numbers indicate significant factors ($p < 0.05$).

References

1. Nuss, J.E.; Wanner, L.M.; Tressler, L.E.; Bavari, S. The osmolyte trimethylamine N-oxide (TMAO) increases the proteolytic activity of botulinum neurotoxin light chains A, B, and E: implications for enhancing analytical assay sensitivity. *J Biomol Screen* **2010**, *15*, 928–936, doi:10.1177/1087057110374996.
2. Jones, R.G.; Ochiai, M.; Liu, Y.; Ekong, T.; Sesardic, D. Development of improved SNAP25 endopeptidase immuno-assays for botulinum type A and E toxins. *J Immunol Methods* **2008**, *329*, 92–101, doi:10.1016/j.jim.2007.09.014.
3. Mizanur, R.M.; Stafford, R.G.; Ahmed, S.A. Cleavage of SNAP25 and its shorter versions by the protease domain of serotype A botulinum neurotoxin. *PLoS One* **2014**, *9*, e95188, doi:10.1371/journal.pone.0095188.

4. Kukreja, R.V.; Sharma, S.K.; Singh, B.R. Molecular basis of activation of endopeptidase activity of botulinum neurotoxin type E. *Biochemistry* **2010**, *49*, 2510–2519, doi:10.1021/bi902096r.
5. Shone, C.C.; Roberts, A.K. Peptide substrate specificity and properties of the zinc-endopeptidase activity of botulinum type B neurotoxin. *Eur J Biochem* **1994**, *225*, 263–270.
6. Schmidt, J.J.; Bostian, K.A. Endoproteinase activity of type A botulinum neurotoxin: substrate requirements and activation by serum albumin. *J Protein Chem* **1997**, *16*, 19–26.
7. Kumar, R.; Kukreja, R.V.; Cai, S.; Singh, B.R. Differential role of molten globule and protein folding in distinguishing unique features of botulinum neurotoxin. *Biochim Biophys Acta* **2014**, *1844*, 1145–1152, doi:10.1016/j.bbapap.2014.02.012.

© 2019 by the authors. Licensee MDPI, Basel, Switzerland. This article is an open access article distributed under the terms and conditions of the Creative Commons Attribution (CC BY) license (http://creativecommons.org/licenses/by/4.0/).
